# Privacy-Preserving Data Aggregation in Two-Tiered Wireless Sensor Networks with Mobile Nodes

**DOI:** 10.3390/s141121174

**Published:** 2014-11-10

**Authors:** Yonglei Yao, Jingfa Liu, Neal N. Xiong

**Affiliations:** 1 School of Computer and Software, Nanjing University of Information Science & Technology, Nanjing 210044, China; E-Mail: ylyao@nuist.edu.cn; 2 Jiangsu Engineering Center of Network Monitoring, Nanjing University of Information Science & Technology, Nanjing 210044, China; E-Mail: jfliu@nuist.edu.cn; 3 School of Computer Science, Colorado Technical University, Colorado Springs, CO 80907, USA

**Keywords:** sensor networks, mobility, data aggregation, privacy protection

## Abstract

Privacy-preserving data aggregation in wireless sensor networks (WSNs) with mobile nodes is a challenging problem, as an accurate aggregation result should be derived in a privacy-preserving manner, under the condition that nodes are mobile and have no pre-specified keys for cryptographic operations. In this paper, we focus on the SUM aggregation function and propose two privacy-preserving data aggregation protocols for two-tiered sensor networks with mobile nodes: Privacy-preserving Data Aggregation against non-colluded Aggregator and Sink (PDAAS) and Privacy-preserving Data Aggregation against Colluded Aggregator and Sink (PDACAS). Both protocols guarantee that the sink can derive the SUM of all raw sensor data but each sensor's raw data is kept confidential. In PDAAS, two keyed values are used, one shared with the sink and the other shared with the aggregator. PDAAS can protect the privacy of sensed data against external eavesdroppers, compromised sensor nodes, the aggregator or the sink, but fails if the aggregator and the sink collude. In PDACAS, multiple keyed values are used in data perturbation, which are not shared with the aggregator or the sink. PDACAS can protect the privacy of sensor nodes even the aggregator and the sink collude, at the cost of a little more overhead than PDAAS. Thorough analysis and experiments are conducted, which confirm the efficacy and efficiency of both schemes.

## 1. Introduction

In recent years, Wireless Sensor Networks (WSNs) have drawn a lot of attention from both academia and industry. Typically, a WSN has a large number of sensor nodes which conduct the sensing operation, process the sensed data and transmit them to the sink. WSNs have very broad applications, for example, military surveillance [1], mobile target tracking [2], environmental monitoring [3], domestics [4], health care [5], to list but a few. WSNs are radically changing the way human beings interact with the environment.

In a WSN, a potentially large number of sensors generate a substantial amount of data. However, sensors are usually resource-limited, suffering from restricted computation, storage, communication resources, and most importantly, battery energy. It is an important challenge to design and develop techniques to efficiently process the data. Fortunately, in many applications, the sink does not need the raw data sensed by certain specific sensors, but instead some statistics. Hence, in-network data aggregation [6] arises to address the resource limitation problem. With data aggregation, either sensors or certain nodes called aggregators aggregate the raw data and/or data received from other sensors, and forward only the aggregated result. This way, the amount of data communicated can be significantly reduced, and as a result, bandwidth consumption and energy depletion are decreased effectively. Typical aggregation functions include SUM, AVERAGE, MAX/MIN, and so on [7]. In this paper, we focus on the additive aggregation function, *i.e.*, SUM.

Another important issue in WSNs are privacy concerns, as wireless sensor network applications are expanding to process increasingly sensitive measurements in everyday life. For example, a Private Households application, as mentioned in CPDA [8], uses wireless sensors placed in houses to collect statistics about water, gas and electricity consumption. The aggregated statistics are very useful for individuals, businesses and government agencies for resource planning and usage advice. However, the individual sensor readings could reveal the daily activities of a household, such as when all family members are absent. Without privacy protection approaches, such applications will not be accepted by people, since participants may not allow tracking their activities. Thus, how to support efficient in-network data aggregation while at the same time preserving data privacy has become an important requirement.

To address the privacy-preserving data aggregation problem, some approaches have been proposed [8–16]. However, most of these schemes focus on data aggregation for WSNs with static topology and homogeneous sensor nodes. In this paper, we address the challenge of designing privacy-preserving protocols for data aggregation in two-tiered mobile WSNs, which are usually formed into cells, each including a static resource-rich aggregator and multiple resource-limited mobile sensor nodes. Such protocols should satisfy the following security and operating requirements: (1) privacy-preserving. The raw sensed data of a sensor node should be protected from being disclosed to any other sensor node, the aggregator, the sink, and an external eavesdropper that can monitor all network traffic; (2) mobility. The large portion of sensor nodes is equipped with some extent of mobility. The privacy of raw data of sensor nodes should be protected even with mobility and dynamic membership; (3) efficiency. Such a protocol is efficient in terms of computation and communication to reduce energy consumption and increase the life time of a WSN. To the best of our knowledge, none of the existing privacy-preserving data aggregation algorithms can completely satisfy these requirements.

To meet the above requirements, this paper presents two privacy-preserving data aggregation protocols called Privacy-preserving Data Aggregation against Aggregator and Sink (PDAAS) and Privacy-preserving Data Aggregation against Colluded Aggregator and Sink (PDACAS), for additive aggregation function in tiered WSNs with mobile sensor nodes.

In PDAAS, each sensor node shares a secret key with the cell header and the sink, respectively. Note that, to support node mobility and save storage, each cell header does not directly share a key with each sensor node, but uses a master key and a pseudorandom function to derive the shared key dynamically when it is required. During the aggregation phase, each sensor node does not send the raw data to the cell header which acts as an aggregator, but instead a perturbed data obtained by processing the raw data two times: in the first time, a keyed value generated from the secret key shared with the sink is added, and in the second time, another keyed value generated from the secret key shared with the aggregator. The cell header removes all the keyed values generated from secrets shared with it, and transmits the intermediate aggregation result to the sink. The sink can recover the final aggregate result by removing all the keyed values generated from secret keys shared with it. This way, the raw data of a node can be protected against other nodes, the aggregator, the sink, and an eavesdropper.

In PDACAS, the aggregators and the sink don't possess any key. Each sensor node is loaded a key ring, which is randomly selected from a large key pool, before deployment. During data aggregation, all the sensor nodes currently in the cell are organized as a conceptual circulation list. Each sensor first perturbs its raw data by multiple keyed values generated from keys in its key ring. Then the intermediate aggregation result will traverse the circulation list two times, each time all the sensor nodes will process it in a one-by-one manner, to remove all the keyed values and submit the intermediate aggregation result to the aggregator. After collecting all the intermediate results, the sink simply adds them up and gets the final aggregation result. With PDACAS, privacy of raw data of a sensor can be protected, even though the aggregator and the sink collude.

We conduct thorough analysis on the proposed schemes in terms of efficacy of privacy preservation. Then we evaluate them in terms of communication and computation overhead. The theoretical analysis and the experimental results demonstrate the efficacy and efficiency of our schemes.

The rest of the paper is organized as follows: Section 2 summarizes the related work. Section 3 describes the network model and assumptions. In Section 4, we present our PDAAS protocol, while Section 5 details the PDACAS protocol, both with theoretical analysis of the privacy-preserving capability. The experiment-based evaluation of the two protocols is presented in Section 6. Section 7 presents our conclusions.

## 2. Related Work

For WSNs, in-network data aggregation is an effective approach to save resources, by aggregating data along the path to the sink and reducing traffic in the network. There has been extensive work that addresses the data aggregation problem in sensor networks, to name a few, [17–20]. However, all these work shares the assumption that sensors are trusted and communications are secure. Thus, privacy issue is not taken into consideration.

Mobility has also been introduced into WSNs, because it is required by some applications, such as wildlife tracking and healthcare. Mobility brings several advantages over traditional WSNs with static nodes [21]: good connectivity, reduced cost, high reliability, and energy efficiency. Extensive work has been conducted to address the problem of routing [22], data gathering [23], key management [24], and so on, for mobile WSNs. However, privacy-preserving data aggregation in mobile WSNs has not been well addressed.

There has been a lot of work on privacy-preserving data aggregation in WSNs. For more technical details on this topic, we refer the reader to some good surveys, such as [25–27]. Here we only briefly summarize the related work. Early work for privacy protection in data aggregation follows a hop-by-hop encryption/decryption approach [9–11]. In these approaches, each intermediate aggregation node has to decrypt the received data, aggregate them and finally encrypt the aggregated result before forwarding it. This approach will incur heavy computation overhead, due to the frequent decryption/encryption, and more seriously, cannot provide data confidentiality at the aggregator nodes.

In [12], homomorphic encryption is employed to support efficient aggregation of encrypted data without decryption involved in the intermediate nodes. However, the scheme does not guarantee the privacy of individual sensed data either against other nodes or against the sink.

The data perturbation technique in [13] inspires PDAAS and PDACAS. In the scheme in [13], during the aggregation phase, each node does not submit its raw data, but rather the sum of its sensed data and a secret value shared with the sink. The sink can recover the final aggregate of sensed values by removing all the secret values. This scheme can protect the privacy of a sensor data against other nodes, but not the sink that can monitor the node's incoming and outgoing traffic.

Two different schemes, CPDA and SMART, are proposed in [8]. In CPDA, sensor nodes are organized into cells, with the cell header acting as the intermediate aggregator. In the intermediate aggregations within cells, each sensor will introduce noise to its raw data. The noises are carefully designed to exploit the cooperation between sensor nodes, such that the accurate aggregated value can be obtained. The computation overhead is heavy in CPDA. In SMART, each sensor divides its sensed data into multiple slices and sends them to selected neighbors in a secure way. Each sensor sums all the received slices and its own slice left, and sends the result to the next aggregator. SMART will incur too much communication overhead.

Recent representative work on privacy-preserving data aggregation for WSNs includes [14–16]. In [14], the authors propose PASKOS and PASKIS, which are also based on the concept of data perturbation [13]. The key idea of both schemes is that, each node computes a perturbed data by adding a secret keyed value to its sensed data and submits only the perturbed data. The keyed values are computed based on keys from a key ring, which is randomly chosen from a key pool. The difference between the two schemes is that, in PASKOS, the sink possesses the whole key pool, whereas in PASKIS, the sink does not possess any key. DyDAP [15] is a dynamic and secure end-to-end data aggregation with privacy function, by employing the similar techniques in [13]. Based on SMART, [16] proposes PEPDA, to reduce collision during data transmission and energy consumption and to compensate loss caused by the collision.

Existing approaches [8–16] are developed for sensor networks with static and homogeneous sensor nodes formed in a flat structure, usually an aggregation tree. In contrast, this paper focuses on privacy-preserving data aggregation in two-tiered WSNs with heterogeneous nodes and dynamic node membership, in which sensor nodes and aggregators do not know each other beforehand. Thus, they have no pre-specified keys for end-to-end encryption. As a result, existing work [8–16] can't be applied directly to this scenario.

## 3. Problem Formulation

### 3.1. Network Model

**Figure 1.**
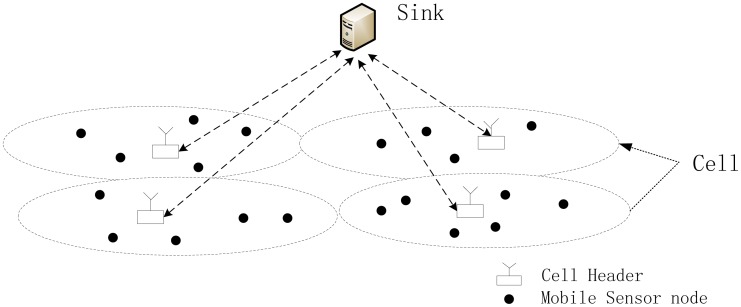
A Two-tiered WSN with Mobile Nodes.

Heterogeneous Wireless Sensor Networks, which include nodes with varying capabilities and usually are organized into a two-tiered structure [23], have drawn a lot of attention to address the fundamental scalability and performance limitations of homogeneous sensor networks [28], in which all sensor nodes have the same capabilities. In a tiered heterogeneous WSN, the lower layer consists of resource-limited sensor nodes performing the sensing task and the upper layer comprises resource-rich nodes acting as intermediate data collectors. In this paper, we consider such two-tiered Wireless Sensor Networks with lower-layered mobile nodes, as shown in Figure 1.

The network region is partitioned into physical cells, each containing a cell header in charge of sensor nodes moving into that cell. The cell headers are resource-rich in terms of storage, energy, communication and computation. These cell headers are all static, *i.e.*, without mobility. They communicate with the sink via relatively long-range, high-rate radios. We assume that there are *m* cell headers, each has a unique ID, denoted as *CH_j_*, *j*∈*{1,2,…,m}*. Usually, *m* is not large.

In contrast, sensor nodes are resource-constrained in storage, energy, and computation. The sensor nodes are mobile and can move among different cells, performing the sensing task to collect data from the environment. They communicate with neighbor nodes or cell headers, but not the sink, via low-power, low-rate, and short-distance radios. Usually, a WSN includes hundreds or even thousands of sensor nodes. We assume that each sensor node also has a unique ID denoted as *s_i_*, *i*∈*{1,2,…,n}*.

A user accesses the network via the sink, collecting aggregated information of the sensed data of all nodes in the network. When queried by the sink, the cell headers act as aggregators, each aggregating the data items collected by the sensors currently in the cell it manages. Typical aggregated information includes SUM, AVERAGE, MIN, MAX, and COUNT. In this paper, we focus on the SUM of sensed raw data of all sensor nodes, based on which other useful aggregated information such as average, count can be computed.

### 3.2. Adversary Model

This paper focuses on thwarting attacks on breaching nodes' data privacy, *i.e.*, preventing the raw data of each sensor node from being disclosed to any other entity. Other important issues, such as data integrity and authenticity, are out of the scope of this paper.

Adversaries may be external or internal. An external adversary is an eavesdropper outside the network, for example, a receiver equipped with powerful antennas. Due to the openness nature of wireless communications, an external eavesdropper can monitor all the traffic across the WSN and intercept transferred messages. As a result, any transferred raw data can be accessed by an external eavesdropper, resulting in the breach of the contributor's data privacy.

Internal adversaries may be malicious or compromised senor nodes, cell headers, or even the sink. The adversary may physically compromise a sensor node and directly access its sensed raw data. In addition, if other sensor nodes use the compromised node as a relay, and transfer raw data or data perturbed by secrets solely shared with the compromised node, their data privacy will be breached. The adversary may also compromise a cell header, and then access data collected from the sensor nodes in the corresponding cell. As with a compromised node, if sensor nodes in the cell transfer raw data or data perturbed by secrets shared with the compromised cell header, their data privacy will be breached. Once the sink is compromised, the adversary can access the key information, if any, and uses it to obtain private data of sensor nodes. In addition, compromised sensor nodes, cell headers, and the sink may collude to attempt deducing sensed raw data of target nodes. For example, if each raw data is perturbed by two secrets, one shared with the cell header and the other with the sink, while the sink and the cell header collude, they can collaboratively recover every raw data.

### 3.3. Design Goals

Our designed approach should simultaneously achieve the following privacy and performance goals:

*Correctness*: The aggregation result should be accurate, *i.e.*, as the same as the result without privacy protection.

*Privacy against the external adversaries*: Our approach should prevent an external eavesdropper from accessing the raw data collected by sensor nodes.

*Privacy against the internal adversaries*: Our approach should also provide raw data privacy against internal adversaries, including malicious or compromised senor nodes, cell headers, or even the sink, considering the situation in which some of them may collude.

*Efficiency*: Above goals on privacy should be achieved with low communication and computation overhead, especially for resource-restricted sensor nodes.

## 4. The PDAAS Protocol

In this section, we present the PDAAS protocol, which can protect the privacy of a node against any other sensor node, the aggregator and the sink, if the aggregator and the sink don't collude. The basic idea is that, each sensor node owns two keys, one shared with the sink and the other shared with the aggregator (cell header). When queried to submit a data, each sensor node computes two keyed values from the two keys, adds the keyed values to the raw data to perturb it, and submits the perturbed data.

### 4.1. Key Distribution

In PDAAS, each sensor node perturbs its raw data using some keyed values before submitting the data, which necessitates a key distribution process. This key distribution process includes two steps: *key pre-distribution* and *keyed value establishment*. Note that, the key distribution process must take the sensor mobility into consideration.

Before deploying the WSN, there is a *key pre-distribution* phase, in which a trusted key server distributes some necessary key information. This key pre-distribution is executed in an offline manner. Specifically, the key server needs to do the following:

(1) For the sink, the key server generates a master key, *MSK*, and loads a *pseudo-random function (PRF)*, *f*.
(2) For each cell header *CH_j_*, the key server also generates a master key, *MCK_j_*, which has an ID same as the cell header's ID, and load the same PRF *f*. As sensor nodes are mobile, each cell header has no idea which sensor node will moves into its cell. In addition, the number of sensor nodes is large. Thus, letting each cell header shares a key with each sensor node is not efficient, as it will consume a lot of storage. In our solution, each cell header is loaded with a master key and a PRF, by which it can generate the shared key with each sensor node
(3) For each sensor *s_i_*, the key server first generates a long-lived key that *s_i_* shares with the sink, *lsk_i_*=*f_MSK_*(*s_i_*) As sensor nodes are mobile and can move between different cells, there is no binding between senor nodes and cell headers. Thus, the key server generates another *m* keys, one for each cell header, $lek_{i}^{j}=f_{MCKj}(si)$ . The key server loads a secure hash function, *Hash*, to the sink, the cell headers, and all the sensor nodes.

Note that, the sink and the cell headers need not to store the keys shared with sensor nodes, as they can reconstruct them when required. The *keyed value establishment* is executed at each sensor node after the WSN is deployed in the interested area and begin to operate, to derive two short-lived keyed values, one shared with the current cell header and the other shared with the sink. These two keyed values will be used in data aggregation.

The sensor nodes collect data from the environment, with some kind of mobility. In order for a sensor node to know the cell it currently stays, each cell header *CH_j_* will periodically broadcast a *hello* message, which includes its ID, to all the sensor nodes currently stayed in the cell. This way, each sensor that just moves in can obtain the ID of the cell header, and choose the corresponding key to be used in data submitting.

When queried by the user, the sink will broadcast a “*data collection*” request message to all the cell headers. This message includes a seed *S_current_* (e.g., a timestamp), which serves as an identification of the current aggregation process. Each cell header forwards this message, plus a nonce $n_{j}^{\text{current}}$ , to all the sensor nodes currently stayed in the cell it manages. After receiving the request, each sensor node *s_i_* will derive two short-lived keyed values, one shared with the sink, $sk_{i}=Hash(lsk_{i}||S_{\text{current}})$ , and the other shared with the cell header *CH_j_*, $ek_{i}^{j}=Hash(lek_{i}^{j}||n_{j}^{\text{current}})$ . Note that, ‖ denotes the concatenating operation.

### 4.2. Data Aggregations

The basic idea of this protocol is that each node uses its two short-lived keyed values to compute a perturbed value, and the cell header can remove one to get the intermediate result, while the sink can remove the other to get the final aggregation result, both without accessing the raw sensor data.

**Algorithm 1.** PDAAS_Sensor_Aggregate.
**Input**: Raw data item *d_i_*; keyed values 
$ek_{i}^{j}$ and *sk_i_***Output**: A perturbed data item *d_i_* with sensor ID**Method**: 1. *d_i_* = *d_i_*; 2. *d_i_* = *d_i_* + *sk_i_*; 3. *d_i_* = *d_i_* + *ek*^j^_i_; 4. Sends <*s_i_*, *d_i_*> to the cell header.


A sensor node *s_i_* follows steps in Algorithm *1*. It simply adds the two keyed values, $ek_{i}^{j}$ and *sk_i_*, to the raw data and gets the perturbed data. Then the sensor sends the perturbed data *d_i_* and its ID to the cell header. Note that, “+” means modular addition as in [13]. That is, the perturbed value is computed as follows:

Perturbed_value=sensed_value+keyed_valuesmodM

We select a sufficiently large value, *i.e.*, M > n*D_max_, for *M*, where *D_max_* is the upper bound for the sensed raw data. This is necessary to remove all keyed values from the aggregation result to obtain the precise sum of the sensed data. For simplicity, we will use “+” and “−” as modular addition and subtraction in this paper.

Each cell header, *CH_j_*, acts as an aggregator of the cell, and follows Algorithm *2*. It first initializes the intermediate aggregation result *D_j_* to be 0, and the set *S_j_* of sensor IDs that contribute to the intermediate aggregation result to be empty. Suppose in current aggregation process, *CH_j_* receives *N_j_* perturbed data items. For each perturbed data item *d_i_* received from sensor node *s_i_*, *CH_j_* first computes the short-lived keyed value shared with *s_i_*, using its master key, sensor ID, the hash function *Hash* and the *PRF f*. Then *CH_j_* subtracts this value from the perturbed data item, and adds the result to *D_j_*.

In addition, *CH_j_* puts the IDs of the sensors that contribute into the set *S_j_*. Finally, *CH_j_* sends <*S_j_*, *D_j_*> to the sink.


**Algorithm 2.** PDAAS_CellHeader_Aggregate.
**Input**: Perturbed data items *d_i_*, i∈{1,2,…*N_j_*}**Output**: An intermediate aggregation result**Method**: 1. *D_j_* = 0; 2. *S_j_* = Ø; 3. For each *d_i_* do 3.
$ek_{i}^{j}=Hash(f_{MCKj}(S_{i})||n_{j}^{\text{current}})$ ;  3.2 *D_j_* = *D_j_* + *d_i_* − 
$ek_{i}^{j}$;  3.3 *S_j_* =*S_j_* ∪ {*s_i_*};  4. Sends <*S_j_*, *D_j_*> to the sink.


The sink follows Algorithm *3* to obtain the final result. It first initializes the aggregation result *D_a_* to be *0*, and the set *S_a_* of IDs that contribute to the aggregation to be empty. For each intermediate aggregation result, the sink adds *D_j_* to *D_a_*, and joins *S_j_* to *S_a_*. Then for each ID in *S_a_*, the sink computes the short-lived keyed value shared with the sensor in current aggregation, using its master key, sensor ID, the hash function *Hash* and the *PRF f*. This value will be subtracted from *D_a_*. Finally, the sink gets the final aggregation result, *D_a_*.

**Algorithm 3.** PDAAS_Sink_Aggregate.
**Input**: <*S_j_*, *D_j_*>, *j* ∈{1,2,…*m*}**Output**: The final accurate aggregation result**Method**: 1. *D_a_* =0; 2. *S_a_* Ø; 3. For each <*S_j_*, *D_j_*> do  3.1 *D_a_* = *D_a_* + *D_j_*;  3.2 *S_a_* = *S_a_* ∪ *S_j_*; 4. For each *s_i_*∈ *S_a_* do  4.1 *sk_i_* =*Hash*(*f_MSK_*(*s_i_*) ‖*S_curret_*);  4.2 *D_a_* =*D_a_* − *sk_i_*;


### 4.3. Analysis of PDAAS

*Correctness*: A data item is perturbed by two keyed values, one shared with the cell header and the other with the sink. At the cell header, it uses the ID of the sensor that collects the data item to derive one of the keyed values, and remove it. At the sink, it derives the other keyed value and removes it. Thus, the sink can obtain an accurate sum of all the data items.

*Privacy*: First, before sending the data item, each sensor node perturbs it using two keyed values. The two keyed values are computed locally and not communicated with any other. Thus, each sensor has a distinct pair of keyed values, which can't be eavesdropped. As a result, PDAAS can protect the data privacy of each sensor node against any other node and an external passive eavesdropper. Second, PDAAS can protect the data privacy of each sensor node against the cell header or the sink, because the data item is perturbed with two keyed values, one shared with the cell header and the other with the sink. If the cell header and the sink don't collude, neither can recover the raw data correctly. Third, PDAAS can protect the data privacy of each sensor node against a powerful external eavesdropper that can compromise a portion of the network (but not the sink and cell headers simultaneously). This is because, without compromising the sink and the cell header at the same time, the adversary can't get both the two keyed values used in perturbing data item. As a result, the adversary can't recover the raw data item. However, if the adversary compromises the sink and a cell header at the same time, or if the sink and a cell header collude, PDAAS fails to protect privacy of any sensor node using the cell header as the aggregator. Let *P_ch_* and *P_sink_* denote the probability that a cell header or the sink is compromised (or intends to collude), and let *P_disclosure_* denote the probability that private data of a sensor node is disclosed:

(1) $$P_{\text{disclosere}}=P_{ch}*P_{\text{sink}}$$

*Efficiency*: In PDAAS, each sensor node keeps a constant (*m* + 1) number of long-lived keys, one shared with the sink, and others shared with cell headers. Thus, the storage overhead per sensor node is bounded by the constant *m* + 1. In each aggregation, each sensor node needs to compute two short-lived keyed values using a hash function, and adds the two keyed values to the raw data item. This will incur a constant computation overhead bounded by *2*. As each sensor node sends its ID and a perturbed data item to the cell header, the communication overhead is also constant, which is log_2_ *n* + log_2_ *M* bits. For the cell header *CH_j_*, it collects *N_j_* perturbed data items, each with an ID, computes *N_j_* keys, and conducts *N_j_* subtractions. Then the cell header sends an intermediate aggregation result and *N_j_* IDs to the sink.

## 5. The PDACAS Protocol

The PDAAS protocol proposed in the previous section cannot protect the privacy of sensor nods in a strong adversary model, where the sink and a cell header collude or are both compromised. To deal with colluded sink and cell headers, this section presents PDACAS.

In PDACAS, each sensor node doesn't share any key with the cell header or the sink, but rather holds a distinct (with high probability) key ring chosen from a large key pool. In data aggregation, each sensor node perturbs its raw data using multiple keyed values generated from keys in its key ring. Then all the sensor nodes collaborate to remove all the keyed values, to get the intermediate aggregation result. This way, even the sink and cell heads collude, they can't get the raw data of sensor nodes. However, overhead incurred by PDACAS is slightly heavier than PDAAS.

### 5.1. Key Distribution

As in PDAAS, before the WSN is deployed, a trusted key server loads some key information to all the sensor nodes. This key pre-distribution scheme is similar to Eschenauer and Gligor's scheme [29], but with a different purpose.

Specifically, the trusted key server first generates a large key pool of *p* keys, KP ={*k*_1_,*k*_2_,…*k_p_*}. Each key in the pool has a unique key ID ranging from *1* to *p*. Then for each sensor *s_i_*, the key server randomly draws *q* keys, *q*≪*p*, from the key pool to establish the key ring of *s_i_*, ${KR}_{i}=\{k_{i}^{1},k_{i}^{2},\ldots k_{i}^{q}\}$ .The key server loads the key ring and the identifiers of all the keys into the memory of *s_i_*. In addition, the key server also loads a *p*-bit bitmap, *bit_i_*, into *s_i_*, with the bits corresponding to the key identifiers in the key ring set to be *1*, and other bits to be *0*. For example, let us suppose *p* = 8, and the key ring of a sensor contains *k_1_*, *k_4_* and *k_6_*, then the bitmap is *10010100*.

As in PDAAS, the key server loads a secure hash function, *Hash*, to all the sensor nodes. Differently from PDAAS, in PDACAS, the trusted key server doesn't load any key information into the sink and the cell headers.

After deployment, all sensor nodes begin to collect data. When a sensor node moves into a new cell, it will receive the periodically broadcasted *hello* message from the cell header, and reply with its ID. Thus, the cell header can obtain an ID set that contains all the IDs of sensor nodes currently in the cell. We use *S_j_* to denote the ID set maintained by cell header *CH_j_*. The cell header will impose an order on all the sensor IDs in *S_j_*; that is, each sensor will have a predecessor and a successor, with the successor of the last be the first one. Conceptually, this means that all the sensor nodes in a cell are organized as a one-way circulation list, with a sensor acting as the head node.

When queried by the user, the sink will broadcast a “*data collection*” request message identified with a seed *S_current_*, to all the cell headers. Each cell header forwards this message to all the sensor nodes in the cell, and each sensor node *s_i_* will compute *q* keyed values: $ek_{i}^{j}=Hash(k_{i}^{j}||S_{\text{current}})$, *1 ≤ j* ≤ *q*. These keyed values will be used to perturb the raw sensed data.

For privacy consideration, a distinct seed for each data collection round is required. The reason is that, as the seed is changed in each round, the keyed values used to perturb the raw data are different. As a result, even though an adversary monitors the communications of the WSN for multiple collection rounds, it is hard to derive the key information of each sensor node. Thus, when a “*data collection*” request message is received, each sensor node will check the seed to make sure that it is a fresh one. In practice, the seeds can be time-based, *i.e.*, the current timestamp. This only requires all the nodes to loosely synchronize with the sink.

### 5.2. Data Aggregations

A cell header *CH_j_* follows Algorithm *4* to get an intermediate aggregation result and send it to the sink. Specifically, each cell header *CH_j_* first broadcasts the ordered sensor ID set *S_j_* to all the sensor nodes in the cell, to let each sensor node know its predecessor and successor. In addition, *CH_j_* initiates the intermediate aggregation result, *D_j_*, to be *0*, and a *p*-bit *0*-string *BIT_j_*. *D_j_* and *BIT_j_* are then sent to the head node of the one-way circulation list. *D_j_* and *BIT_j_* will traverse the one-way circulation list two times, each time all the sensor nodes will process them in a one-by-one manner. Afterwards, the cell header will receive the intermediate aggregation result, and report it to the sink.

**Algorithm 4.** PDACAS_CellHeader_Aggregate.
**Input**: None**Output**: An intermediate aggregation result *D_j_***Method**: 1. Broadcasts the ordered ID set *S_j_* to all the sensor nodes in the cell; 2. *D_j_* = 0; 3. Initiates a *p*-bit bitmap *BIT_j_*, with each bit to be *0*; 4. Sends *D_j_* and *BIT_j_* to the head node in *S_j_*; 5. Waits for *D_j_* and *BIT_j_* to traverse the one-way circulation list of sensor nodes two times; 6. receives *D_j_*; 7. Sends *D_j_* to the sink.


For each sensor node *s_i_*, it initiates a counter, *c_i_*, to be 0, and follows the steps in Algorithm *5*. Note that, *c_i_* is used to indicate how many times that *s_i_* has received *D_j_* and *BIT_j_*.

When receiving *D_j_* and *BIT_j_*, *s_i_* first increments *c_i_* by *1*. Then according to different *c_i_*, *s_i_* will take different actions. If *c_i_* equals to *3*, which indicates that *D_j_* and *BIT_j_* have traversed the one-way circulation list of sensor nodes two times, *s_i_* will transmit *D_j_* to the cell header.

If *c_i_* equals to *1*, which means that *s_i_* is processing *D_j_* and *BIT_j_* for the first time, *s_i_* will perturb its raw data item and adds the perturbed data to *D_j_*. In the perturbation, *s_i_* checks each *k*-th (1 ≤ k ≤ p) bit of *BIT_j_* and *bit_i_*: If both equal to *1*, which means that there is some sensor node which holds a same key, thus a same keyed value, with *s_i_*, and has used this keyed value to perturb its raw data, *s_i_* will subtract the keyed value from *d_i_*, and clear the *k*-th bit of *BIT_j_*; if the *k*-th bit of *bit_i_* is *1* while the *k*-th bit of *BIT_j_* is *0*, *s_i_* will add the keyed value generated from this key to *d_i_* and set the *k*-th bit of *BIT_j_*. The perturbed data item is added to *D_j_*, and *s_i_* will send *D_j_* and *BIT_j_* to its successor.

If *c_i_* equals to *2*, which means that *s_i_* is processing *D_j_* and *BIT_j_* for the second time, *s_i_* will remove keyed values generated from some of its keys. It checks each *k*-th (1 ≤ k ≤ p) bit of *BIT_j_* and *bit_i_*: If both equal to 1, *s_i_* will subtract the corresponding keyed value from *D_j_* and clear the *k*-th bit of *BIT_j_*. Then *s_i_* sends *D_j_* and *BIT_j_* to its successor.

Note that, a sensor may send data to the successor directly, if both sensor nodes are in the communication range of each other; or indirectly through the cell header otherwise.

After receiving all the intermediate aggregation results from the cell headers, the sink retrieves the final aggregation result simply by summing them up, as shown follows:

(2) $$D_{a}=\sum_{j=1}^{m}D_{j}$$

**Algorithm 5.** PDACAS_Sensor_Aggregate.
**Input**: *D_j_*; *BIT_j_*; *bit_i_*; *S_j_*; raw data *d_i_*; keyed values 
$ek_{i}^{j}$, *1 ≤ j* ≤ *q***Output**: Altered *D_j_* and *BIT_j_***Method**: 1. *c_i_* = *c_i_* + 1; 2. if *c_i_* = 3  transmit *D_j_* to the cell header *CH_j_*;  return 3. if *c_i_* = 1  3.1 **for** (int *k* = 1; *k* <= p; *k*++)    **if** both the *k*th bit of *BIT_j_* and *bit_i_* are *1*s     
$d_{i}=d_{i}-ek_{i}^{j}$     Set the *k*th bit of *BIT_j_* to be 0;    **else if** the *k*th bit of *bit_i_* is 1     
$d_{i}=d_{i}+ek_{i}^{j}$     Set the *k*th bit of *BIT_j_* to be 1;  3.2 *D_j_* = *D_j_* + *d_i_*;  3.3 transmits *D_j_* and *BIT_j_* to the successor;  3.4 return 4. if *c_i_* = 2  4.1 **for** (int *k* = 1; *k* <= p; *k*++)    **if** both the *k*th bit of *BIT_j_* and *bit_i_* are *1*s     *D_j_* = *D_j_* − 
$ek_{i}^{j}$;     Set the *k*th bit of *BIT_j_* to be 0;  4.2 transmits *D_j_* and BITj to the successor;  4.3 return


### 5.3. Analysis of PDACAS

*Correctness*: In data aggregation, all the sensor nodes are organized as a one-way circulation list, and *D_j_* will traverse this list two times.

When *D_j_* traverses the one-way circulation list for the first time, each sensor will perturb its data item by keyed values generated from its keys, and add the perturbed data to *D_j_*. In raw data perturbation, sensor node *s_i_* checks each keyed value it possesses. If a keyed value has been used by some other sensor node (because that sensor node and *s_i_* share a common key to generate the same keyed value), *s_i_* will subtract this keyed value; otherwise, this keyed value is added. When the first travel of *D_j_* is finished, *D_j_* equals to the sum of all the raw data items and some keyed values.

When *D_j_* traverses the one-way circulation list for the second time, each sensor node will remove the keyed values generated from some of its keys, which have been added but not removed in *D_j_*'s first travel. Thus, all the keyed values will be removed, and the cell header can get the accurate aggregate of all sensed values.

*Privacy*: First, PDACAS can protect privacy of any sensor node against an external passive eavesdropper, as the raw data is perturbed by *q* keyed values. Second, PDACAS can protect privacy of any node's raw data against the cell header and the sink, even they collude, because the cell headers and the sink don't possess any key, and can't remove the keyed values used in perturbing sensor data. Third, PDACAS can protect privacy of any node against certain number of colluded other sensor nodes, or an external and active attacker who has compromised certain number of sensor nodes, with a high probability. This is because, a sensor node uses all the keys in its key ring when perturbing its raw data item. As a result, to recover a node's raw data, the adversary has to obtain the whole key ring of targeted node. By choosing appropriate parameters *p* and *q*, the probability that a sensor's key ring is covered by the union of certain number of other key rings is negligible, as shown in the following.

Assuming that *t* nodes are compromised by the adversary, but the target node *s_i_* is not compromised. Without loss of generality, the key rings of the *t* compromised nodes are denoted as *KR_j_*, j∈ {1,2,…*t*}. Because PDACAS and PASKOS [21] both use key rings in data perturbation, following the analysis of PASKOS in [21], we have:

(3) $$\begin{array}{l} P_{\text{disclose}}=P\{KR_{i}\subseteq \overset{t}{\underset{j=1}{\cup }}KR_{j}\}\\ =1-P\{\exists k_{i}^{1}\in KR_{i},1\leq l\leq q,k_{i}^{l}\notin \overset{t}{\underset{j=1}{\cup }}KR_{j}\}\\ =1P\{k_{i}^{1}\notin \overset{t}{\underset{j=1}{\cup }}KR_{j},\vee \ldots \vee k_{i}^{q}\notin \overset{t}{\underset{j=1}{\cup }}KR_{j}\}\\ =\sum_{k=0}^{q}{\left(-1\right)}^{k}\left(\begin{array}{c} q\\ k \end{array}\right)\left(\frac{\left(\begin{array}{c} p-k\\ q \end{array}\right)}{\left(\begin{array}{c} q\\ k \end{array}\right)}\right) \end{array}$$

For example, let us suppose the key pool size is *p* = 100 and the key ring size is *q* = 4. The probability that a raw data of a sensor node can be recovered by some certain 10 and 20 nodes is 1.11% and 6.36%, respectively. Thus, even the key pool is not large and the key ring of each sensor node is small, the probability that a raw data of a sensor node can be recovered is acceptable.

*Efficiency:* In PDACAS, each sensor node stores a *p*-bit bitmap, a key ring comprising *q* keys, and *q* key identifiers. Thus, the storage overhead per sensor node is *p* + q*L_key_ + q*log_2_p bits, where *L_key_* denotes the length of a key. In addition, each sensor node needs to compute *q* keyed values, and performs 2*q additions or subtractions in the worst case. Thus, the computation overhead in PDACAS is bounded by *q*.

As communication is concerned, each sensor node sends *D_j_* and *BIT_j_* to its successor two times. As a result, the communication overhead incurred by sending is 2*(p + *log_2_M)* bits. In addition, each sensor node will receive *D_j_* and *BIT_j_* from its predecessor two times, resulting in a 2*(p + *log_2_M)* bits receiving overhead.

## 6. Performance Evaluation

In this section, we first present numerical results of privacy-preserving efficacy of the proposed two protocols, in terms of probability that a sensor node's raw data might be recovered. Then we present simulation-based evaluation of the efficiency of the two proposed protocols, in terms of energy consumption in the privacy-preserving operations.

### 6.1. Efficacy

In PDAAS, a sensor's raw data is disclosed when a cell header and the sink are both compromised (or both intend to collude). The probability of this disclosure is simple and is given in Equation (1). For simplicity, we set *P_ch_* = *P_sink_= p_d_*. Thus, the disclosure probability of a sensor node's raw data is a quadratic function of *p_d_*. In a situation where the probability that a cell header and the sink are both compromised (or both intend to collude) is expected to be low, PDAAS can protect the data privacy of each sensor in an effective way.

In PDACAS, a sensor's raw data is disclosed when the following two events occur simultaneously:

(1) Some other nodes are compromised, and
(2) The target node's key ring is covered by the union of the key rings of all the compromised nodes.

The probability of the first event is a simple *t*th-order function of the probability that a sensor node is compromised, where *t* is the number of compromised sensor nodes. We ignore to discuss this probability, as it is straightforward.

The probability of the second event is given in Equation (2), which is much complicated. In practice, WSNs have different scales, depending on the different applications. We compute the probabilities with different key pool and key ring sizes. Network operators can choose appropriate parameters based on the scale of their WSNs.

**Figure 2.**
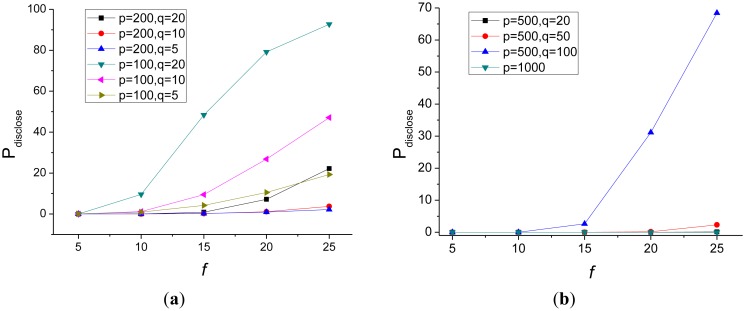
Theoretical value of *P_disclose_* in PDACAS with (**a**) small key pool and key ring and (**b**) large key pool and key ring.

Figure 2a presents the theoretical values of this probability when the key pool and the key ring of each sensor are small. Specifically, we set the key pool size *p* = 100 and *p* = 200, respectively. Then for key ring size *q* = 5, *q* = 10, and *q* = 20, we compute *P_disclose_* for different number of compromised sensor nodes. As shown in Figure 2, for a certain *p*, smaller key ring size indicates better privacy, which is consistent with the intuition that, the number of shared keys in any two key rings is smaller when the key ring size is smaller. For a certain *q*, larger key pool size can provide better privacy protection, because the percentage of shared keys in any two key rings is smaller when the key pool is larger. For certain *p* and *q*, the more other sensor nodes are compromised, the worse privacy it can provide. This is easy to understand, because when more other sensor nodes are compromised, the probability that the target node's key ring is covered by the union of key rings of the compromised nodes increases.

For small key pool size and key ring size, when the number of compromised nodes increases, the probability that the raw data of the target node might be recovered will also increase dramatically. As a result, small key pool size and key ring size are only applicable to WSNs in which the number of sensor nodes in a cell at certain time is not large.

Figure 2b presents the probability that a target node's key ring is covered by the union of key rings of certain compromised other nodes, when the key pool size and the key ring size are relatively large.

When the key pool size *p* = 1000, *p_dislose_* is negligible for any key ring size less than 100. If we set the key pool size *p* = 500, and choose a small key ring size, for example, not more than 50, *p_dislose_* is also acceptable. So, if the WSN is dense and at certain period, there are a large number of sensor nodes moving into a cell, to provide a good privacy with PDACAS, we should choose a large key pool and relatively small key rings.

### 6.2. Efficiency

To evaluate the computation and communication overhead of the two proposed protocols, we conduct simulations. The simulation environment is TOSSIM under TinyOS. We consider a WSN with 500 mobile nodes which move uniformly at random among 20 cells, each with a fixed cell header acting as an aggregator. On average, there are 25 sensor nodes stayed in a cell in certain time period. The length of the sensor ID is⌈log_2_ 500⌉ = 9 bits. The communication range for each node is set to 30 m*,* a typical one in an indoor environment.

We set the upper bound for the sensed value *D_max_* = 500, which is sufficiently large for most WSN applications, as WSNs are deployed to collect data from the environment, such as temperature, humidity, CO_2_ concentration, and so on. Thus, the upper bound for the perturbed data item can be computed as 500 × 500 = 250,000, and the maximal length of a perturbed data item is ⌈log_2_ 250000⌉ = 18 bits. We choose HMAC as the hash function, and set the size of the key to be 128 bits.

For PDAAS, the overhead per sensor node is constant. The computation overhead includes two hash operations and two addition operations, and the communication overhead is incurred by sending a sensor ID and a perturbed data item. The overhead in terms of energy consumption on MICAz and TelosB is shown in Table 1.

On the MICAz platform, the energy cost is larger than TelosB. However, even on MICAz, the overhead in terms of energy cost for privacy-preserving operations is only about 2000 μJ, which has little impact on lifetime of sensor nodes.

**Figure 3.**
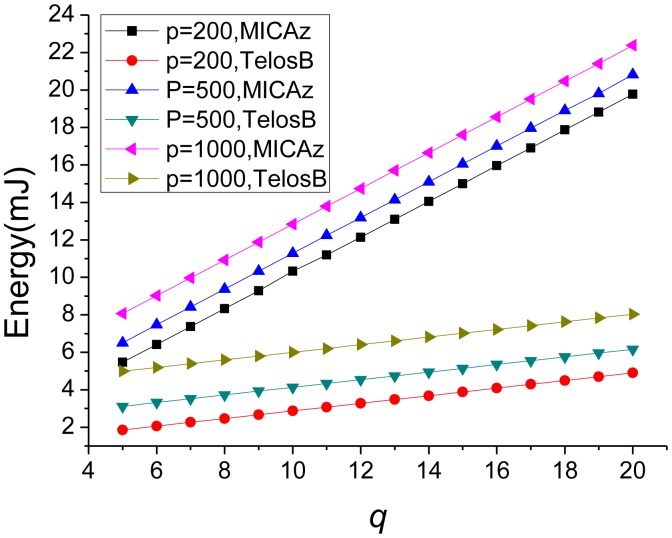
PDACAS Overhead.

For PDACAS, the overhead incurred per sensor node in privacy-preserving operations is a function of *p* and *q*. Figure 3 summarizes the overhead in terms of energy cost of PDACAS on MICAz and TelosB, with different key pool size *p* and key ring size *q*.

As shown in Figure 3, the energy consumption increases with the increase of the key pool size *p*. The reason is that, each sensor node will receive and send a *p*-bit bitmap twice in the data aggregation process. If *p* increases, a sensor node will consume more energy on communication. In addition, when *p* is given, the energy cost increases roughly linearly as *q* increases. This is because, as described in Section 5.3, the computation overhead includes *q* hash operations and *2q* additions/subtractions, a linear function of *q*. From Figure 3, we can also see that, even with a large key pool, the total energy consumed on computation and communication for privacy protection is small, and will not post a great threat on the lifetime of a sensor node.

**Figure 4.**
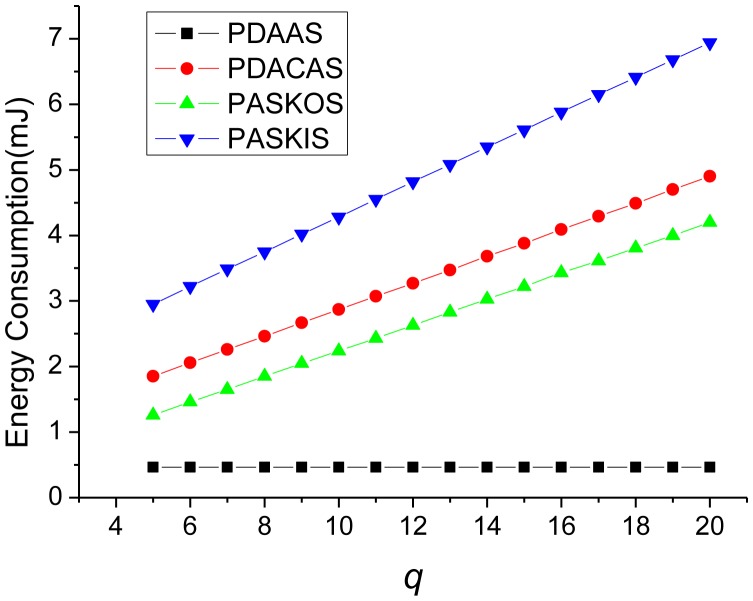
Energy Consumption Comparison.

We compare the proposed two protocols with PASKOS and PASKIS [14], because all of them are based on the concept of data perturbation [13], even though PASKOS and PASKIS are designed for sensor networks with static and homogeneous sensor nodes. In PASKOS and PASKIS, all sensors are homogeneous and are formed into an aggregation tree. In the comparison, for simplicity, we set the branch factor of each non-leaf node in the aggregation tree of PASKOS and PASKIS to be 3. We set the key pool size *p* = 200 and vary the key ring size *q*. The result on TelosB platform is shown in Figure 4.

As in Figure 3, when *p* is given, the communication overhead is fixed, *i.e.*, two receiving and two sending, each involving a *p*-bit bitmap and a perturbed data item. However, the computation overhead is a linear function of *q*, *i.e.*, *q* hash operations and *2q* additions/subtractions. As a result, the total energy cost increases linearly as *q* increases.

Our PDAAS protocol incurs the least overhead, because the overhead per sensor node is constant. In PDAAS, the computation overhead per sensor node involves only two hash operations and two additions, and the communications overhead is only *1* message sending.

Our PDACAS protocol incurs a little more overhead than PASKOS. The reason is that, in PDACAS each sensor node needs to receive and send a *p*-bit string and the perturbed data items twice, resulting in more communication overhead.

PASKIS incurs the most overhead. In PASKIS, an aggregation request broadcast containing a *p*-bit bitmap needs to be sent to each sensor node first. Then each non-leaf sensor node collects multiple perturbed data items plus a *p*-bit bitmap from its children. In addition, the data perturbation process involves a multiply operation. As a result, the PASKIS protocol will incur more overhead than the other three ones.

### 7. Conclusions

In this paper, we address the privacy protection problem for data aggregation in two-tiered WSNs with fixed aggregators and mobile sensor nodes. Two privacy protection protocols, namely PDAAS and PDACAS, are presented. These two protocols provide an effective protection against both external eavesdroppers and internal malicious entities. Specifically, PDAAS can protect privacy of a sensor node against other nodes, the aggregator, the sink, and an eavesdropper. PDACAS can protect privacy of a sensor's raw data even the sink and the aggregator collude, at the cost of a slightly increased overhead. Thorough analysis and experimental evaluation confirm the efficacy and efficiency of both protocols. In the future, an evaluation of the two protocols on real deployments is an aspect that requires more in-depth investigation. In addition, solutions to privacy-preserving data aggregation for other types of WSNs, e.g., tiered WSNs in which both aggregators and sensor nodes are mobile, have yet to be proposed.

## Figures and Tables

**Table 1.** PDAAS OVERHEAD.

**Operation**	**MICAz**	**TelosB**
Computation	1890 *μJ*	392 *μJ*
Communication	69 *μJ*	73 *μJ*
